# Cannabis Use Is Associated With Recurrence After Primary Spontaneous Pneumothorax

**DOI:** 10.3389/fsurg.2021.668588

**Published:** 2021-05-25

**Authors:** Connor J. Wakefield, Christopher W. Seder, Andrew T. Arndt, Nicole Geissen, Michael J. Liptay, Justin M. Karush

**Affiliations:** Department of Cardiovascular and Thoracic Surgery, Rush University Medical Center, Chicago, IL, United States

**Keywords:** spontaneous pneumothorax, pneumothorax recurrence, marijuana, chest tube thoracostomy, video-assisted thoracic surgery

## Abstract

**Purpose:** Primary spontaneous pneumothorax (PSP) is a frequently encountered entity that carries a high rate of recurrence. The current study aims to investigate if cannabis use at time of initial PSP is associated with disease recurrence.

**Methods:** Patients presenting with PSP between 2010 and 2018 at a single institution were identified. Exclusion criteria included secondary pneumothorax, severe chronic lung disease, lung cancer, and lost to follow-up. Patients were compared relative to their cannabis usage with Fisher's exact test, Wilcoxon rank-sum test, and logistic regression.

**Results:** Overall, 67 patients (53 male) met inclusion criteria with a median body mass index (BMI) of 21.5 kg/m^2^ (IQR 19.1–25.2) and age of 34 years (IQR 22–53). Initial treatment consisted of chest tube in 42 patients (63%), video-assisted thoracoscopic surgery wedge resection in 19 patients (28%), and observation in 6 patients (9%). Cannabis users (*n* = 28; 42%) had a higher rate of tobacco use (79 vs. 38%; *p* = 0.005), lower BMI [21.0 kg/m^2^ (IQR 18.3–23.1) vs. 22.2 kg/m^2^ (IQR 19.9–28.6), *p* = 0.037], and were more likely to require intervention at first presentation compared with non-marijuana users. Cannabis use was associated with PSP recurrence when adjusting for tobacco use, BMI, and height (OR 1.85, 95% CI 1.38–18.3, *p* = 0.014).

**Conclusion:** There is a high rate of cannabis usage in patients presenting with PSP. Cannabis usage is associated with PSP recurrence and eventual need for operative intervention.

## Introduction

Primary spontaneous pneumothorax (PSP) is a common entity that carries a high rate of recurrence. PSP has an incidence of 7.4–18 per 100,000 people and is typically seen in tall, thin, young adult males between the ages of 20 and 30 years (1, 2). PSP represents ~85% of spontaneous pneumothorax cases encountered while the other are due to iatrogenic causes, trauma, or chronic lung disease (3). Patients presenting with PSP often complain of acute onset shortness of breath and moderate–severe pleuritic chest pain. Although the symptom severity of PSP varies with the degree of lung collapse, large pneumothoraces can present with respiratory distress and hemodynamic compromise warranting emergent intervention. It has been estimated that recurrence of pneumothorax can be seen in 17–54% of patients that present with a new PSP, and recurrence rates as high as 20% have been seen in patients who undergo surgical intervention (2, 4, 5). Tobacco smoking is considered a major risk factor for development of PSP, conferring a 9 and 22-fold increased relative risk in women and men who smoked, respectively (6). This increased risk is thought to be due to cigarette-induced respiratory bronchiolitis and emphysematous changes in small airways (7, 8).

Daily cannabis use is also thought to contribute to lung pathology, although the risk is considered lower than that seen with daily tobacco use (9). Cannabis remains one of the most commonly used illicit drugs in developed countries with a lifetime prevalence of 37.1 and 46.4% in female and males, respectively (10). Isolated reports of pneumothorax and pneumomediastinum have been attributed to the deep and prolonged inspiration practices used by cannabis users (11–14). Furthermore, there is a limited number of reports suggesting a link between cannabis use and PSP (15–18). The hypothesis of this study is that cannabis use is associated with an increased rate of PSP recurrence.

## Materials and Methods

Institutional Review Board approval was obtained before any research activities (IRB # 19111702-IRB01) and informed consent was not required. Patients who presented with PSP between 2010 and 2018 at a university-based, tertiary medical center were identified. Recurrence was defined as development of ipsilateral PSP after complete resolution of initial PSP. Complete resolution of initial PSP was confirmed through documented resolution on radiologic imaging at >30 days' duration between episodes of pneumothorax. Exclusion criteria included traumatic and iatrogenic causes of pneumothorax, severe chronic lung disease, lung cancer, previous thoracic surgery, and <60 days of follow-up. Severe chronic lung disease was defined as those with pulmonary function testing before presentation demonstrating a forced expiratory volume (FEV) of <50% of predicted values or FEV/force vital capacity <0.7. Cannabis use was collected retrospectively from the patient history at time of presentation and recurrence within electronic medical records.

Demographic characteristics, patient-reported use of tobacco and recreational substances, treatment received, and outcome data were retrospectively collected. Binary and categorical variables were summarized by frequency (%). Continuous variables were summarized by median and interquartile range. Univariate analysis of categorical and dichotomous variables was performed with χ^2^ test and Fisher's exact test. Univariate analysis of non-parametric continuous variables was performed with Wilcoxon rank-sum test. Multivariate analysis was performed with logistic regression and the outcome variable was pneumothorax recurrence. Predictor variables included in the model were those determined to be associated with pneumothorax recurrence in previous reports or variables found to be significantly associated with PSP recurrence (*p* < 0.05) on univariate analysis.

## Results

Overall, 109 medical charts were reviewed and 67 patients met inclusion criteria with a median body mass index (BMI) of 21.5 kg/m^2^ [interquartile range (IQR) 19.1–25.2], height of 177.9 cm (IQR 167.6–185.4), age of 34 years (IQR 22–53), and mean follow-up of 671.5 days (190.5–990.5). Most patients were male [79% (53/67)] and 28 patients (42%) reported cannabis use at presentation. There was no difference in age, race, gender, or chronic lung disease between cannabis and non-cannabis users (Table 1). Recurrence of PSP occurred in 26 (39%) of patients, 15 (54%) of which were cannabis users and 11 (28%) who were not cannabis users (*p* = 0.03).

**Table 1:** Patient characteristics.

	**All patients**	**Marijuana users**	**Non-marijuana user**	***p*-value**
	**(*n* = 67)**	**(*n* = 28)**	**(*n* = 39)**	
Age, years; median (IQR)	34 (22.0–52.5)	37 (22.0–48.3)	31 (20.0–53.0)	0.57
Gender, Male	53 (79)	21 (75)	32 (82)	0.55
BMI, kg/m^2^; median (IQR)	21.5 (19.1–25.2)	21 (18.3–23.1)	22.2 (19.9–28.6)	**0.04**
Height, cm, median (IQR)	177.8 (167.6–185.4)	179.1 (169.6–185.4)	177.5 (167.6–182.9)	0.63
**Race or ethnic group**				0.31
Caucasian	31 (46)	10 (36)	21 (53)	
African American	23 (34)	12 (43)	11 (28)	
Hispanic	4 (6)	1 (3)	3 (8)	
Asian	1 (2)	0 (0)	1 (3)	
Other	8 (12)	5 (18)	3 (8)	
**Smoker**				**0.01**
Never	30 (45)	6 (21)	24 (61)	
Past	14 (21)	7 (25)	7 (18)	
Current	23 (34)	15 (54)	8 (21)	
Pack years among current and former smokers, median (IQR)	9.5 (5.0–18.3)	7.5 (5–15)	10 (6.9–18.5)	0.561
History of chronic lung disease	6 (9)	3 (11)	3 (8)	0.67

*All data presented as n (%), unless otherwise indicated. BMI, body-mass index; IQR, interquartile range*.

*Bold values refers to significant for p < 0.05*.

Cannabis use was associated with concurrent tobacco use (*p* = 0.005), PSP recurrence (*p* = 0.012), and eventual need for surgical intervention at any time throughout disease course (*p* = 0.049). There was no difference in amount of cigarette usage as measured in pack years between those who did and did not use cannabis. There was no difference in the need for operative intervention at initial presentation between cannabis smokers and non-cannabis smokers. Initial treatment for PSP consisted of chest tube in 42 patients (63%), chest tube followed by VATS blebectomy and mechanical pleurodesis in 19 patients (28%), and supplemental oxygen in 6 patients (9%) (Table 2). Six (15%) of non-cannabis users were managed with supplemental oxygen at initial presentation while no cannabis users were (*p* = 0.03). Previous tobacco use and current tobacco without concurrent cannabis use was not associated with PSP recurrence. Height and BMI also were not found to be associated with PSP recurrence. Multivariate analysis demonstrated that marijuana use was significantly associated with PSP recurrence when adjusting for concurrent tobacco use, height, and BMI (OR 1.85, 95% CI 0.34–3.37, *p* = 0.016; Table 3).

**Table 2:** Treatment and outcomes.

	**All patients**	**Marijuana users**	**Non-marijuana user**	***p*-value**
	**(*n* = 67)**	**(*n* = 28)**	**(*n* = 39)**	
Initial treatment^*^				
Supplemental oxygen	6 (9)	0 (0)	6 (15)	**0.03**
CT	42 (63)	18 (64)	24 (62)	0.82
CT + VATS	19 (28)	10 (36)	9 (23)	0.26
**Need for operative intervention at anytime**				
CT + VATS	41 (61)	21 (75)	20 (51)	**0.04**
**Outcomes**				
Recurrence				**0.03**
Ipsilateral	26 (39)	15 (54)	11 (28)	
Time to recurrence, median months (IQR)	11 (2.0–39.0)	12 (3.0–84.0)	7.8 (1.6–15.3)	0.25

*All data presented as n (%), unless otherwise indicated. CT, chest thoracostomy; CT + VATS, chest thoracostomy + video assisted thoracoscopic surgery; IQR, interquartile range*.

* *Treatment performed within first hospitalization for initial presentation*.

*Bold values refers to significant for p <0.05*.

**Table 3:** Logistic regression model for PSP recurrence.

**Treatment Method**	**Regression coefficient (*B*)**	**Odds ratio exp (*OR)***	***p*-value**	**95% CI**
Marijuana use	1.61	4.50	**0.014**	[1.38, 18.3]
Current tobacco use	−0.4	0.98	0.946	[0.28, 3.30]
Height	0.05	1.04	0.093	[0.99, 1.10]
BMI	0.10	1.11	**0.039**	[1.01, 1.22]

*CI, Confidence interval*.

## Discussion

The current study is the largest series to date investigating the rate of recurrence in patients with PSP. We found that those presenting with PSP and who report current cannabis use are at an increased risk of pneumothorax recurrence when adjusted for other known risk factors. Cannabis use has been hypothesized to impact the rate of recurrence in patients presenting with PSP although the literature remains inconclusive. Through both animal and human studies, cannabis use was reported to negatively affect airway physiology (9, 19). Specifically, habitual cannabis use alone has been found to cause macroscopic and microscopic injury to large airways leading to an increased likelihood of chronic bronchitis (9). In addition, cannabis use has also been reported to promote increased lung volumes and airway resistance secondary to the common inhalation practices of prolonged and deep inspiration (9). Consistent with these findings, a report of 10 habitual cannabis smokers found all had evidence of bullous lung disease on CT imaging (20). It is through these observations that cannabis use may have a role in development of PSP. However, many report the risk of cannabis usage to be lower than that of tobacco because there is no direct association between cannabis use and development of lung cancer (9). Also, reports are mixed on the association of cannabis use and development of PSP and subsequent risk of pneumothorax recurrence. A limited number of case reports and small case series have suggested a possible link between daily cannabis use and development of PSP and pneumomediastinum. One large series of 416 patients that presented with PSP found that combined tobacco and cannabis usage significantly increased the risk of developing PSP (21). However, the effect of cannabis usage, in the absence of other inhalants, on the rate of PSP recurrence is less understood.

The findings of this study have many implications as cannabis is a frequently used recreational drug. With cannabis becoming legalized in many states, there is concern that more people, including youth, will adopt cannabis smoking (22). In fact, the USA saw an increase in self-reported past-30-day use of cannabis, from 18 to 20.2% between the years of 2002 and 2016, in college students (22). It is unknown how this increasing trend of cannabis usage will effect disease prevalence. This study did not include minors, thus further studies will need to be conducted in this age group to fully understand their risks and disease courses.

There are notable limitations of this study. First, this is a retrospective study that was not able to verify cannabis usage through toxicology screening or detail the method and quantify of cannabis use. Patients were evaluated for substance abuse based on history and presentation inquiry. Because of this, we feel self-reported rates of cannabis use may in fact be an underestimate of actual usage. This is also possible because marijuana use was illegal at the time of data collection.

In conclusion, we report a high rate (43%) of cannabis usage in our cohort of patients that presented with PSP. This is well-above the national average of reported past-30-day cannabis usage (8.81%) among persons 12 years of age and higher (23). Cannabis usage was associated with concurrent tobacco use, PSP recurrence, and need for VATS pleurodesis. We feel it is important to educate patients presenting with PSP on the possible effects cannabis inhalation may have on their disease process.

## Data Availability Statement

The raw data supporting the conclusions of this article will be made available by the authors, without undue reservation.

## Ethics Statement

The studies involving human participants were reviewed and approved by Rush University Medical Center Institutional Review Board. Written informed consent for participation was not required for this study in accordance with the national legislation and the institutional requirements.

## Author Contributions

CW, CS, and JK contributed to the conceptual design of the study. CS and JK contributed to the project administration, supervision of the study, and wrote the first draft of the manuscript. CS, AA, NG, ML, and JK contributed to data curation. CW organized the database and performed statistical analysis. All authors contributed to manuscript revision, read, and approved the submitted version.

## Conflict of Interest

The authors declare that the research was conducted in the absence of any commercial or financial relationships that could be construed as a potential conflict of interest.

## References

[1] Baumann MH Noppen M. Pneumothorax. Respirology. (2004) 9:157–64. 10.1111/j.1440-1843.2004.00577.x 15182264

[2] Tulay CM Ozsoy IE. Spontaneous pneumothorax recurrence and surgery. Indian J Surg. (2015) 77 (Suppl. 2):463–5. 10.1007/s12262-013-0876-6 26730046 PMC4692894

[3] Bobbio A Dechartres A Bouam S Damotte D Rabbat A Régnard JF. Epidemiology of spontaneous pneumothorax: gender-related differences. Thorax. (2015) 70:653–8. 10.1136/thoraxjnl-2014-206577 25918121

[4] Sadikot RT Greene T Meadows K Arnold AG. Recurrence of primary spontaneous pneumothorax. Thorax. (1997) 52:805–9. 10.1136/thx.52.9.805 9371212 PMC1758641

[5] Massongo M Leroy S Scherpereel A Vaniet F Dhalluin X Chahine B. Outpatient management of primary spontaneous pneumothorax: a prospective study. Eur Respir J. (2014) 43:582–90. 10.1183/09031936.00179112 23766331

[6] Bense L Eklund G Wiman LG. Smoking and the increased risk of contracting spontaneous pneumothorax. Chest. (1987) 92:1009–12. 10.1378/chest.92.6.1009 3677805

[7] Cheng YL Huang TW Lin CK Lee SC Tzao C Chen JC. The impact of smoking in primary spontaneous pneumothorax. J Thorac Cardiovasc Surg. (2009) 138:192–5. 10.1016/j.jtcvs.2008.12.019 19577078

[8] Weissberg D Refaely Y. Pneumothorax: experience with 1,199 patients. Chest. (2000) 117:1279–85. 10.1378/chest.117.5.1279 10807811

[9] Tashkin DP. Effects of marijuana smoking on the lung. Ann Am Thorac Soc. (2013) 10:239–47. 10.1513/AnnalsATS.201212-127FR 23802821

[10] Agrawal A Budney AJ Lynskey MT. The co-occurring use and misuse of cannabis and tobacco: a review. Addiction. (2012) 107:1221–33. 10.1111/j.1360-0443.2012.03837.x 22300456 PMC3397803

[11] Hazouard E Koninck JC Attucci S Fauchier-Rolland F Brunereau L Diot P. Pneumorachis and pneumomediastinum caused by repeated muller's maneuvers: complications of marijuana smoking. Ann Emerg Med. (2001) 38:694–7. 10.1067/mem.2001.118016 11719752

[12] Goodyear K Laws D Turner J. Bilateral spontaneous pneumothorax in a cannabis smoker. J R Soc Med. (2004) 97:435–6. 10.1258/jrsm.97.9.435 15340025 PMC1079587

[13] Birrer RB Calderon J. Pneumothorax, pneumomediastinum, and pneumopericardium following valsalva's maneuver during marijuana smoking. N Y State J Med. (1984) 84:619–20. 6335229

[14] Miller WE Spiekerman RE Hepper NG. Pneumomediastinum resulting from performing valsalva maneuvers during marihuana smoking. Chest. (1972) 62:233–4. 10.1378/chest.62.2.233 5050235

[15] Tan C Hatam N Treasure T. Bullous disease of the lung and cannabis smoking: insufficient evidence for a causative link. J R Soc Med. (2006) 99:77–80. 10.1177/014107680609900220 16449781 PMC1360494

[16] Taylor DR Hall W Thoracic Society of Australia and New Zealand. Respiratory health effects of cannabis: position statement of the thoracic society of australia and new zealand. Intern Med J. (2003) 33:310–3. 10.1046/j.1445-5994.2003.00401.x 12823677

[17] Hancox RJ Poulton R Ely M Welch D Taylor DR McLachlan CR. Effects of cannabis on lung function: a population-based cohort study. Eur Respir J. (2010) 35:42–7. 10.1183/09031936.00065009 19679602 PMC3805041

[18] Shah A Paramlal M. The importance of an illicit drug history in the evaluation of suspected spontaneous pneumothorax. BMJ Case Rep. (2011) 2011:3693. 10.1136/bcr.01.2011.3693 22693289 PMC3132656

[19] Tashkin DP. Airway effects of marijuana, cocaine, and other inhaled illicit agents. Curr Opin Pulm Med. (2001) 7:43–61. 10.1097/00063198-200103000-00001 11224724

[20] Hii SW Tam JD Thompson BR Naughton MT. Bullous lung disease due to marijuana. Respirology. (2008) 13:122–7. 10.1111/j.1440-1843.2007.01186.x 18197922

[21] Hedevang Olesen W Katballe N Sindby JE Titlestad IL Andersen PE Ekholm O. Cannabis increased the risk of primary spontaneous pneumothorax in tobacco smokers: a case-control study. Eur J Cardiothorac Surg. (2017) 52:679–85. 10.1093/ejcts/ezx160 28605480

[22] Odani S Soura BD Tynan MA Lavinghouze R King BA Agaku I. Tobacco and marijuana use among us college and noncollege young adults, 2002-2016. Pediatrics. (2019) 144:e20191372. 10.1542/peds.2019-1372 31712275

[23] National Survey on Drug Use and Health. 2014-2016 Substate Estimates of Substance Use and Mental Illness. Rockville, MD: National Survey on Drug Use and Health (2018).

